# Infidelity among Physicians

**Published:** 1883-07

**Authors:** 


INFIDELITY AMONG PHYSICIANS.—Dr. Henry Gibbons, Sr. in an article published in the *Pacific Medical and Surgical Journal,* entitled "Religious Elements of Human Character," writes as follows:

" Physicians have long been charged by professors of religion with what they choose to call " infidelity." The charge has grown out of the well-known liberality of the members of the profession, and their habit of tolerating all modes of faith. Mingling socially with all classes of society and with individuals holding all shades of belief, physicians in the exercise of their calling see farther into men's hearts than the average observer. They discover more imperfection in the so-called good, and more virtue in the opposite class, than are generally recognized. They take human nature at mean and they exercise charity. Judging men by their conduct rather than by their creeds, they attach but little importance to peculiarities of belief. They do not meddle with the faith of their patients, but leave them to derive from belief and from ceremonial observances all comfort possible. They sometimes make enemies of ministers of religion by excluding their well meant exercise from the sick chamber where the patient's life is at hazard, and where any disturbance or excitement, religious though it be, might turn the scale and open the gates of death. Physicians, in short, judge by the rule of the poet:

" He can't be wrong whose life is in the right."

" They believe in the biblical declaration that " this is true religion and acceptable to God the father, to visit the widow and the fatherless in their affliction and to keep oneself unspotted from the world."

" But what shall be said of these men who, by conversation, writing and lecturing, devote themselves to the destruction of the religious sentiment in youth, and to the robbery, as it may be called, of the faith of men ? That such faith is blind and without evidence, and that it is linked with superstition, is not alone reason enough to attempt its eradication, without substituting anything better. Shall I take away from my crippled brother the staff which gives uncertain support, without supplying him with a better ? If I find him shivering with cold with only a ragged coat for protection, shall I persuade him to throw away the garment without providing him with a warmer ? A low and superstitious faith is better than none ; as the frail staff is better than no support, and the ragged garment better than nakedness."

